# Draft Genome Sequences of *Penicillium* spp. from Deeply Buried Oligotrophic Marine Sediments

**DOI:** 10.1128/MRA.01613-18

**Published:** 2019-02-07

**Authors:** Morgan S. Sobol, Tatsuhiko Hoshino, Taiki Futagami, Fumio Inagaki, Brandi Kiel Reese

**Affiliations:** a Department of Life Sciences, Texas A&M University—Corpus Christi, Corpus Christi, Texas, USA; b Kochi Institute for Core Sample Research, Japan Agency for Marine-Earth Science and Technology (JAMSTEC), Nankoku, Japan; c Education and Research Centre for Fermentation Studies, Faculty of Agriculture, Kagoshima University, Kagoshima, Japan; Georgia Institute of Technology

## Abstract

Here, we report genome sequences of two *Penicillium* isolates from below the seafloor of the oligotrophic South Pacific Gyre. These genomes are the first reported for fungi from deeply buried marine sediment. Both genomes will provide valuable information regarding the role of fungi and carbon cycling in the energy-limited subsurface biosphere.

## Abstract (precis)

Here, we report genome sequences of two *Penicillium* isolates from below the seafloor of the oligotrophic South Pacific Gyre. These genomes are the first reported for fungi from deeply buried marine sediment.

## ANNOUNCEMENT

Recent advances in molecular and culturing techniques have verified the presence and activity of eukaryotic fungi in deeply buried marine sediment. However, the physiological and metabolic capabilities of fungi in the deep marine subsurface are still poorly understood (1, 2). Whole-genome sequencing is invaluable to delineate the role that marine subsurface fungi play in global biogeochemical cycles and their survivability in an energy-limited system. Here, we present draft genome sequences of two *Penicillium* isolates from the oligotrophic sediments of the South Pacific Gyre. Isolates of *Penicillium* sp. SPG-F1 and SPG-F15 were collected during the Integrated Ocean Drilling Program Expedition 329 at 124 m below the seafloor (mbsf) and 12 mbsf, respectively. Sediment was enriched using 10% marine broth media (Difco 2216; BD Diagnostics), and the fungi were subsequently isolated and maintained in potato dextrose broth (Difco 254920; BD Diagnostics) at ambient temperature prior to nucleic acid extraction.

High-quality genomic DNA was extracted by following a cetyltrimethylammonium bromide (CTAB)-based method (3) with modifications. An Illumina paired-end library was prepared following the manufacturer's protocol (TruSeq DNA PCR-Free Sample Preparation Guide, part number 15036187 Rev. A; http://research.lunenfeld.ca/ngs/truseq_dna_pcrfree_sampleprep_guide_15036187_a.pdf) to generate 101-bp length paired-end reads. The isolates were sequenced using a HiSeq 2000 sequencer (Illumina, San Diego, CA) at Macrogen in Seoul, South Korea, which produced 162,517,156 reads for *Penicillium* sp. SPG-F15 and 149,610,352 reads for *Penicillium* sp. SPG-F1.

Low-quality reads (quality score [*Q*] of <20) were removed, and Illumina adapters were trimmed from the reads using Trim Galore! The average read length after trimming was 101 bp for both isolates. The genomes were assembled *de novo* using SPAdes v3.11.0 (4). In SPAdes, the coverage cutoff was set to 10× and the careful mode turned on to reduce the number of mismatches and errors. The genome of *Penicillium* sp. SPG-F15 was assembled into 348 contigs totaling 36.13 Mbp in length with a 46.52% G+C content. The assembly coverage was 436×. Isolate *Penicillium* sp. SPG-F1 was assembled into 457 contigs totaling 32.06 Mbp with a 48.26% G+C content. The assembly coverage was 446×. The lengths and G+C contents of both genomes were comparable to those of other *Penicillium* species of continental origin (5). The genomes contained approximately 98% complete orthologs according to Benchmarking Universal Single-Copy Orthologs (BUSCO) software (6).

Repetitive elements were identified and masked with RepeatModeler v1.0.11 (7) and RepeatMasker v4.0.7 (8). A total of 3,550,914 bp (9.82%) of repetitive elements were masked in *Penicillium* sp. SPG-F15, whereas *Penicillium* sp. SPG-F1 had 928,433 bp (2.90%) masked. For annotation, a MAKER2 pipeline was used (9, 10) that incorporated *ab initio* gene predictions from both Augustus (11) and GeneMark-ES (12). A total of 11,243 protein-coding sequences were found in *Penicillium* sp. SPG-F15 and 11,581 were found in *Penicillium* sp. SPG-F1, which is consistent with those in other *Penicillium* species (5). A phylogenomics approach was used to identify both organisms as *Penicillium*. Single-copy homologues from both genomes in this study were clustered and compared with *Penicillium* and *Aspergillus* genomes (downloaded from NCBI GenBank and the Joint Genome Institute) using GET_homologues (13). The ortholog search was carried out with OrthoMCL using a default cutoff of 1e−05.

The predicted protein sequences were given to Blast2GO version 5.1 for putative gene ontology assignment, classifying enzyme codes, and Kyoto Encyclopedia of Genes and Genomes mapping (14). The proteins were also annotated with the Carbohydrate-Active enZYmes (CAZy) database (15). Genes involved in hydrocarbon, lignin, lignocellulose, and carbonate degradation were present in both genomes, but a more detailed analysis of these metabolic pathways will be assessed in a future publication. Both genomes will serve as important resources for understanding the influence that fungi have on recalcitrant carbon degradation in the marine subsurface.

### Data availability.

The genomic sequences and assemblies have been deposited at GenBank under BioProject accession numbers PRJNA435885 (*Penicillium* sp. SPG-F15) and PRJNA435890 (*Penicillium* sp. SPG-F1). This announcement represents the first versions of both genomes.
